# HOXA4, down-regulated in lung cancer, inhibits the growth, motility and invasion of lung cancer cells

**DOI:** 10.1038/s41419-018-0497-x

**Published:** 2018-04-27

**Authors:** Shaofei Cheng, Fengying Qian, Qin Huang, Lirong Wei, Yawen Fu, Yuzhen Du

**Affiliations:** 1 Department of Thoracic-cardiovascular Surgery, Shanghai Jiao Tong University Affiliated Sixth People’s Hospital, Shanghai, China; 2 Department of Laboratory Medicine, Shanghai Jiao Tong University Affiliated Sixth People’s Hospital, Shanghai, China; 3 Department of Pathology, Shanghai Jiao Tong University Affiliated Sixth People’s Hospital, Shanghai, China

## Abstract

The involvement of HOXA4 in colorectal cancer and epithelial ovarian cancer has been reported. Although it has been reported that the *Hoxa4* gene is involved in the patterning of the mouse lung during embryonic development, little is known about the biological functions of HOXA4 in lung cancer. In the current study, HOXA4 expression was down-regulated in lung cancer tissues when compared with non-cancerous tissues. HOXA4 expression was associated with tumor size, TNM stage, lymph node metastasis and prognosis. Bioinformatics analysis revealed that HOXA4 expression was negatively correlated with cell cycle, metastasis, and the Wnt signaling pathway. Moreover, HOXA4 overexpression in lung cancer cell lines suppressed cell proliferation, migration, and invasion. HOXA4 decreased the protein expression levels of β-catenin, Cyclin D1, c-Myc and Survivin, indicating the inhibition of Wnt signaling. HOXA4 significantly increased the protein and mRNA levels of glycogen synthase kinase-3β (GSK3β) by promoting its transcription. Furthermore, inhibition of GSK3β by LiCl abolished the suppression of cell growth, migration, and invasion mediated by HOXA4. Overexpression of HOXA4 in xenograft tumors also decreased tumor growth and Wnt signaling. Collectively, these data suggest that HOXA4 is a potential diagnostic and prognostic marker in lung cancer, and its overexpression could inhibit lung cancer progression in part by promoting GSK3β transcription.

## Introduction

Lung cancer represents the leading cause of cancer-related mortality in the world^1^. The most frequent type of lung cancer is non-small cell lung cancer (NSCLC), which accounts for ~85% of lung cancer cases^1^. The overall survival for most patents with lung cancer is relatively low^2^, mainly because of the lack of obvious initial symptoms and effective therapy. Recently, studies have identified several lung cancer-related pathways, including the epidermal growth factor receptor (EGFR)^3,4^, p16^INK4^/Cyclin D1/Rb^5^ and Wnt signaling pathways^6^. Therapy targeting these pathways has provided a broad prospect for the treatment of lung cancer^7,8^.

HOXA4 belongs to the Homeobox (HOX) gene family, which is characterized by the presence of a 183-base pair DNA sequence (homeobox) that encodes a highly conserved homeodomain. HOX genes encode transcription factors that control cell differentiation and embryonic development by binding to the promoters of various target genes and regulating their expression^9,10^. Previous studies have investigated the regulation and expression of the *Hoxa4* gene in mouse embryos^11–13^ and suggested that the *Hoxa4* gene is involved in the patterning of the mouse lung^14^. Accumulated evidence has indicated the abnormal expression of development-associated genes in cancers and their contributions to carcinogenesis. HOXA4 is reportedly overexpressed in colorectal cancer^15^ and epithelial ovarian cancer^16^. Further study revealed that HOXA4 suppresses migration in ovarian cancer cell lines via β1 integrin^17^. Although other members of the HOX gene family, such as HOXA5, HOXA10, HOXB3, HOXB4, and HOXC6^18,19^, have been found to be overexpressed in lung cancer tissues compared with normal tissues, little is known about the expression and biological function of HOXA4 in lung cancer.

In this study, we demonstrated that HOXA4 was down-regulated in lung cancer tissues compared with non-cancerous tissues. We then performed functional characterization of HOXA4 in human lung cancer cell lines with HOXA4 overexpression or silencing. Our study showed that HOXA4 overexpression repressed the growth, motility and invasion of lung cancer cells and inhibited the Wnt pathway. Our findings suggest that HOXA4 may be a potential therapeutic target for lung cancer.

## Results

### HOXA4 expression is decreased in human lung cancer tissues

**Fig. 1:**
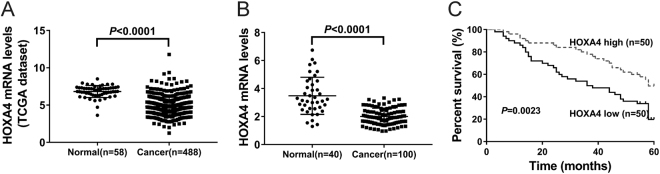
Decreased expression of HOXA4 in human lung cancer tissues was associated with poor prognosis. **a** The expression levels of HOXA4 were analyzed in lung cancer and normal lung tissues from the TCGA lung cancer dataset. **b** HOXA4 mRNA expression was analyzed in lung cancer and normal lung tissues from our hospital. **c** Kaplan–Meier survival curve analysis of patients with high or low HOXA4 expression

First, we analyzed HOXA4 expression in human lung cancer tissues by using a dataset downloaded from The Cancer Genome Atlas project (TCGA, https://tcga-data.nci.nih.gov/tcga/). Figure 1a shows that HOXA4 expression levels were decreased in lung cancer tissues (*n* = 488) compared with normal lung tissues (*n* = 58) (4.81 ± 0.06 vs. 6.82 ± 0.10, *P* < 0.0001). Further, quantitative reverse transcription-PCR (qRT-PCR) analysis was performed to examine the expression of HOXA4 in lung cancer samples and adjacent normal tissues obtained from patients at our hospital (Fig. 1b). We found that HOXA4 mRNA expression was lower in tumor tissues (*n* = 100) than in normal lung tissues (*n* = 40) (2.01 ± 0.06 vs. 3.49 ± 0.21, *P* < 0.0001). These data suggest the clinical significance of HOXA4 in lung cancer diagnosis.

### Decreased expression of HOXA4 is associated with tumor size, TNM stage, lymph node metastasis and poor prognosis in lung cancer

We then examined the correlation between HOXA4 expression and clinical pathological features. The 100 lung cancer patients were split into two groups: the HOXA4 high expression group and HOXA4 low expression group. As analyzed by Fisher’s exact test (Table 1), we found that HOXA4 expression in lung cancer was significantly associated with tumor size (*P* = 0.0262), TNM stage (*P* = 0.0088) and lymph node metastasis (*P* = 0.0076). However, HOXA4 expression was not correlated with age (*P* = 0.6845), gender (*P* = 0.4230), or smoking status (*P* = 0.5269) in lung cancer.

**Table 1:** Correlation of HOXA4 expression with patients’ features

Variables	All cases	HOXA4 mRNA		*P* value
		Low (*n*=50)	High (*n*=50)	
Age at surgery				
<55	41	22	19	0.6845
≥55	59	28	31	
Gender				
Male	53	24	29	0.4230
Female	47	26	21	
Smoking status				
Smoker	34	19	15	0.5269
Non-smoker	66	31	35	
Tumor size				
<5 cm	44	16	28	0.0262*
≥5 cm	56	34	22	
TNM stage				
I+II	46	16	30	0.0088**
III	54	34	20	
Lymphnode metastasis				
Absent	60	23	37	0.0076**
Present	40	27	13	

Fisher’s exact test; ^*^P < 0.05, ^**^P < 0.01.

Kaplan–Meier analysis and the log-rank test were then conducted to assess the relationship between HOXA4 expression and the outcomes of lung cancer patients after surgery. The overall survival (OS) curves (Fig. 1c) showed that patients with lower HOXA4 expression levels had poorer OS (*P* < 0.01). These results suggest that HOXA4 expression may be a novel prognostic marker for lung cancer.

### Analysis of HOXA4-associated pathways

**Fig. 2:**
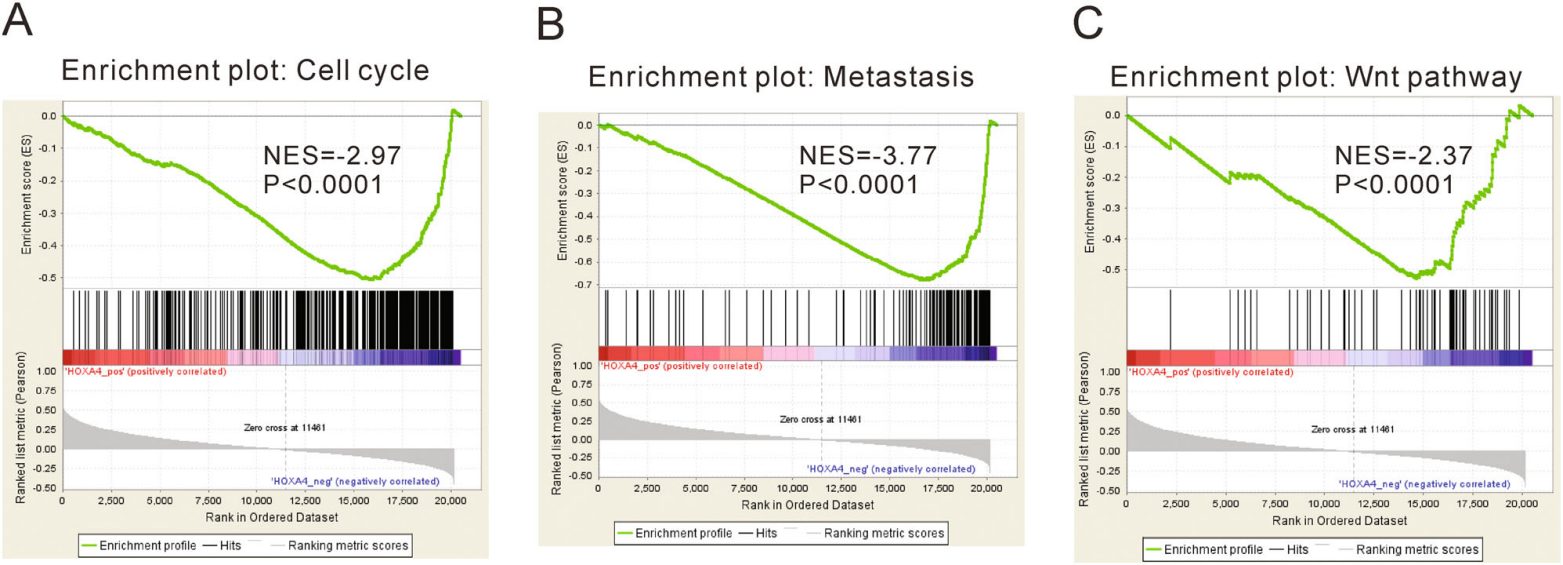
GSEA analysis showed that HOXA4 was negatively associated with cell cycle (**a**), metastasis (**b**), and the Wnt signaling pathway (**c**) in the TCGA lung cancer samples

Gene set enrichment analysis (GSEA) was performed to evaluate pathways that were associated with HOXA4 expression in the TCGA lung cancer samples. The results revealed that HOXA4 expression was negatively correlated with cell cycle, metastasis and the Wnt signaling pathway (Fig. 2), which implied that HOXA4 may affect the growth, invasion and migration of lung cancer.

### Manipulation of HOXA4 expression in lung cancer cell lines

**Fig. 3:**
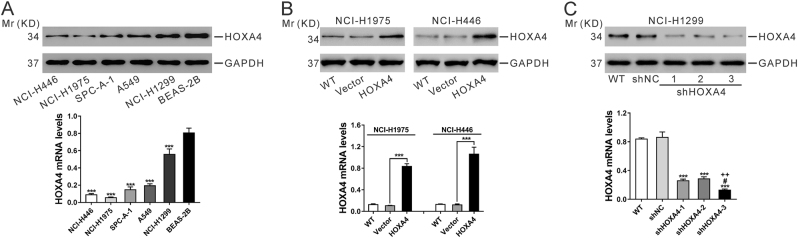
Manipulation of HOXA4 expression in lung cancer cell lines. **a** Protein (top panel) and mRNA (bottom panel) expression of HOXA4 in 5 lung cancer cell lines and the BEAS-2B cell line. GAPDH was used as an internal control. **b** NCI-H1975 and NCI-H446 cells were infected with HOXA4-expressing lentivirus or control vector lentivirus (Vector), and HOXA4 protein (top panel) and mRNA (bottom panel) expression were analyzed at 48 h after viral infection. Wild-type cells (WT) served as a negative control. ***P < 0.001. **c** NCI-H1299 cells were infected with HOXA4 shRNA lentivirus (shHOXA4-1, 2, and 3) or control shRNA lentivirus (shNC). HOXA4 protein (top panel) and mRNA (bottom panel) expression were analyzed at 48 h post viral infection. ***P < 0.001 vs. shNC, ^#^*P* < 0.05 vs. shHOXA4-1, ^++^*P* < 0.01 vs. shHOXA4-2

To investigate the biological implications of HOXA4 in lung cancer, we first analyzed the mRNA and protein expression levels of HOXA4 among 5 lung cancer cell lines and 1 normal lung epithelium cell line via qRT-PCR and western blot. The expression of HOXA4 was significantly down-regulated in lung cancer cell lines, especially in the NCI-H1975 and NCI-H446 cell lines (Fig. 3a). We then used lentiviral transduction to overexpress HOXA4 in NCI-H1975 and NCI-H446 cells and knocked down HOXA4 expression in NCI-1299 cells, which had relatively high expression of HOXA4. qRT-PCR and western blot analyses were conducted at 48 h after transduction. As illustrated in Fig. 2b, c, vector or control shRNA (shNC) viral transduction had no effects on HOXA4 expression. The mRNA and protein levels of HOXA4 were significantly increased in cells transfected with HOXA4 virus compared with wild-type cells (WT) or cells transfected with vector virus (Fig. 3b). Three different shRNAs (shHOXA4-1, 2, and 3) remarkably reduced the levels of HOXA4 in NCI-1299 cells among which shHOXA4-3 (referred to as shHOXA4) had the best knockdown efficiency (Fig. 3c) and was selected for the following functional assays.

### HOXA4 inhibits growth and promotes apoptosis of lung cancer cells in vitro

**Fig. 4:**
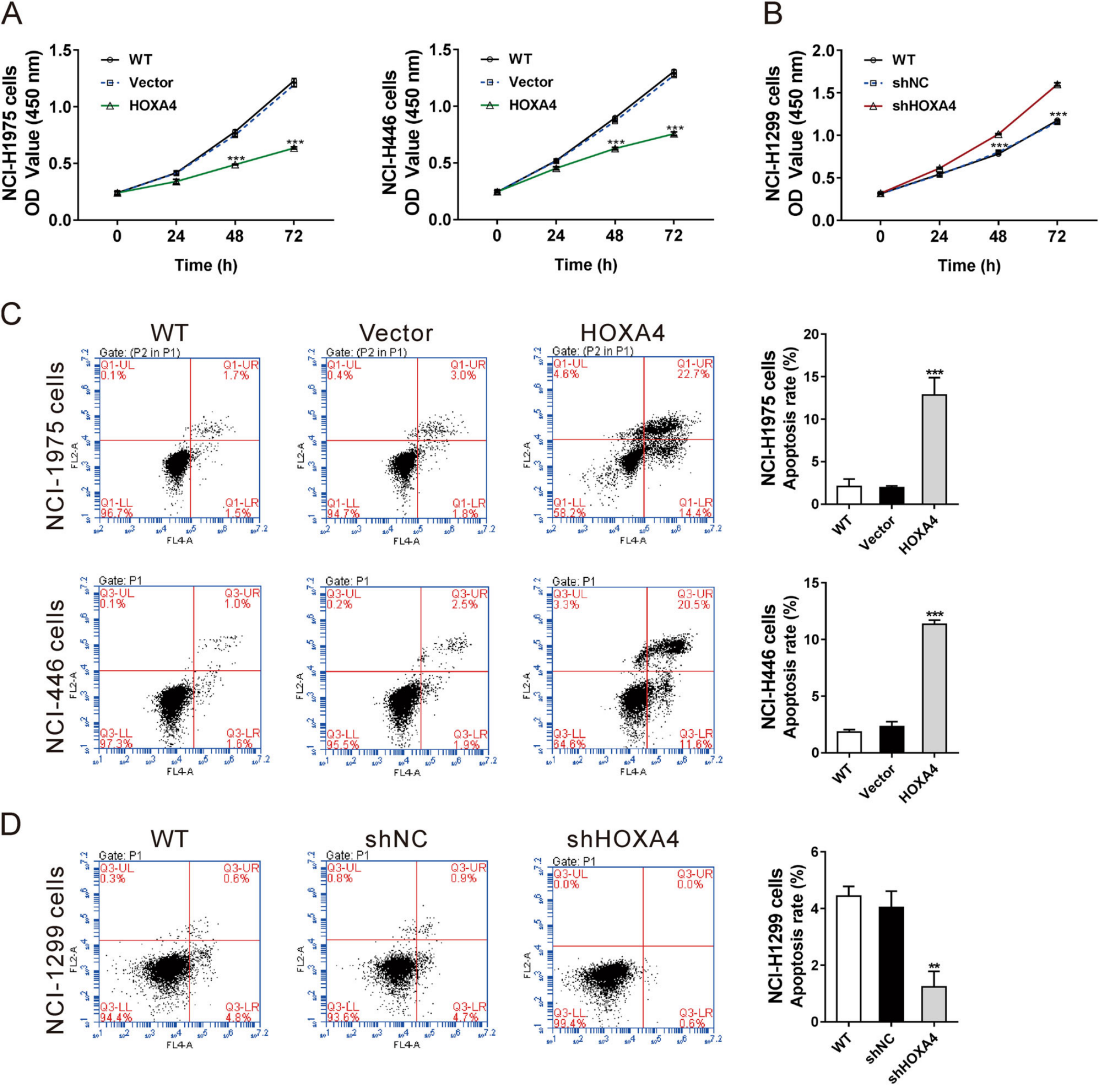
HOXA4 inhibited the growth and promoted the apoptosis of lung cancer cells in vitro. **a**CCK-8 assays were used to determine the proliferation of NCI-H1975 and NCI-H446 cells in which HOXA4 had been overexpressed. ***P < 0.001 vs. vector. **b** CCK-8 assays were performed to determine the proliferation of NCI-1299 cells in which HOXA4 had been silenced. ***P < 0.001 vs. shNC. **c** Annexin V/PI staining was performed to determine the apoptosis of NCI-H1975 and NCI-H446 cells in which HOXA4 had been overexpressed. ***P < 0.001 vs. vector. **d** Annexin V/PI staining was performed to determine the proliferation of NCI-1299 cells in which HOXA4 had been silenced. ***P < 0.001 vs. shNC

Cell Count Kit-8 (CCK-8) assays were carried out to explore the functions of HOXA4 regarding lung cancer cell proliferation. Ectopic expression of HOXA4 significantly inhibited cell growth in NCI-H1975 and NCI-H446 cells compared with the controls (WT and vector) (Fig. 4a). In contrast, knocking down HOXA4 expression promoted cell proliferation in NCI-H1299 cells (Fig. 4b). Annexin V staining followed by cytometry analysis was performed to explore the effects of HOXA4 on lung cancer cell apoptosis. HOXA4 overexpression significantly promoted cell apoptosis in NCI-H1975 and NCI-H446 cells compared with controls (WT and vector) (Fig. 4c), whereas knocking down HOXA4 expression had the opposite effect on NCI-H1299 cells (Fig. 4d). These data imply that HOXA4 may act as a tumor suppressor involved in the inhibition of cancer cell proliferation and promotion of cancer cell apoptosis.

### HOXA4 suppresses lung cancer cell migration and invasion

**Fig. 5:**
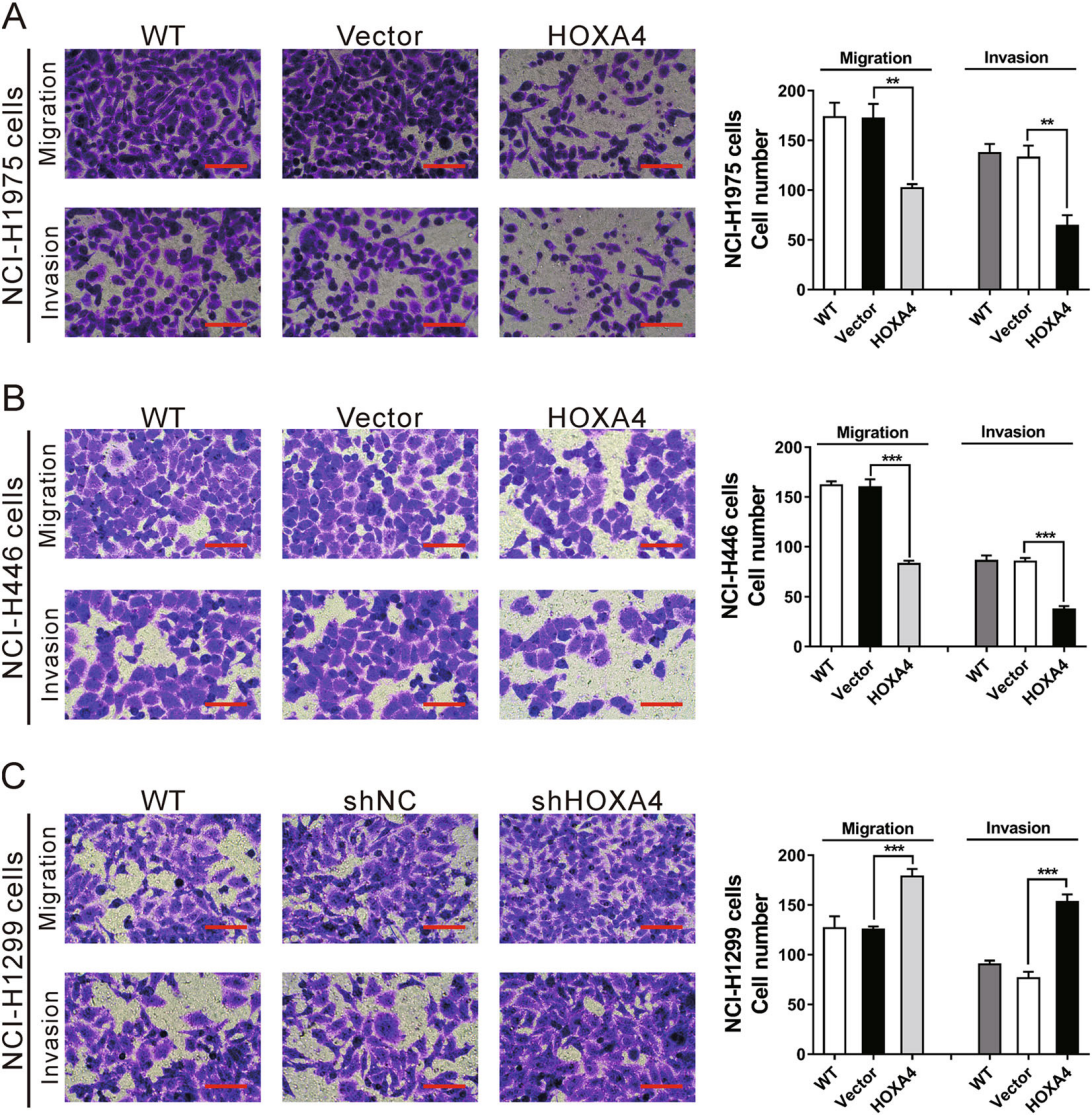
HOXA4 suppressed lung cancer cell migration and invasion. Transwell assays with or without Matrigel were performed to determine the invasion and migration abilities of NCI-H1975 (**a**) and NCI-H446 (**b**) cells with HOXA4 overexpression and NCI-1299 cells (**c**) with HOXA4 silencing. Magnification: ×200. Scale bars=100 μm. ***P < 0.001

Transwell assays with or without Matrigel were performed to determine the influence of HOXA4 on lung cancer cell invasion and migration, respectively. Compared with the controls, the ectopic expression of HOXA4 in NCI-H1975 and NCI-H446 cells led to a notable decrease in cell migration and invasion (Fig. 5a, b). However, knocking down HOXA4 expression in NCI-H1299 cells promoted cell migration and invasion (Fig. 5c). These data indicate that HOXA4 suppresses lung cancer cell migration and invasion.

### HOXA4 suppresses the Wnt signaling pathway in lung cancer cells

**Fig. 6:**
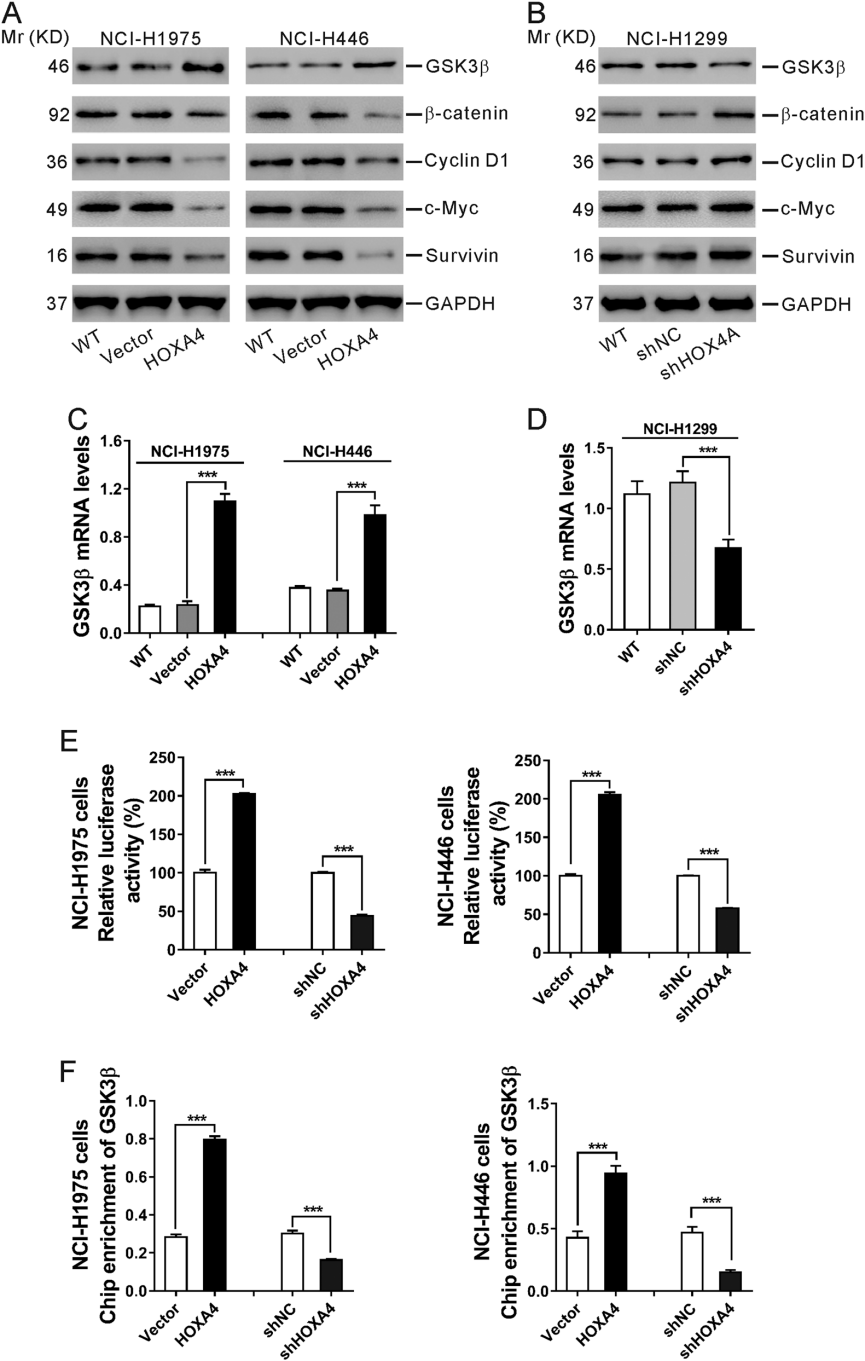
HOXA4 suppressed the Wnt signaling pathway in lung cancer cells. **a**, **b** Western blot analysis of GSK3β, β-catenin, Cyclin D1, c-Myc and Survivin in NCI-H1975 and NCI-H446 cells with HOXA4 overexpression (**a**) and NCI-H1299 cells with HOXA4 silencing (**b**). GAPDH was used as a loading control. **c**, **d** qRT-PCR analysis of GSK3β mRNA levels in NCI-H1975 and NCI-H446 cells with HOXA4 overexpression (**c**) and NCI-H1299 cells with HOXA4 silencing (**d**). **e** A luciferase reporter assay was performed to evaluate GSK3β promoter activity in NCI-H1975 and NCI-H446 cells with HOXA4 overexpression or silencing. **f** ChIP-qPCR for the GSK3β promoter in NCI-H1975 and NCI-H446 cells with HOXA4 overexpression or silencing. ***P < 0.001

Activation of the Wnt pathway can induce cell proliferation and invasion and is likely to be crucial in promoting carcinogenesis^20^. Considering that HOXA4 expression was negatively correlated with the Wnt signaling pathway (Fig. 2c), we supposed that HOXA4 could suppress Wnt signaling. Expression of the major component in the Wnt pathway (glycogen synthase kinase-3β [GSK3β]) and downstream effectors of the Wnt pathway (β-catenin, Cyclin D1, c-Myc, and Survivin) was assessed by western blot in lung cancer cells in which HOXA4 had been overexpressed or knocked down. As shown in Fig. 6a, the ectopic expression of HOXA4 in NCI-H1975 and NCI-H446 cells resulted in a notable increase in GSK3β protein levels and an obvious decrease in β-catenin, Cyclin D1, c-Myc, and Survivin protein levels. Knocking down HOXA4 expression in NCI-H1299 cells showed the opposite effect.

Similarly, qRT-PCR results revealed that the mRNA level of GSK3β was significantly increased following the overexpression of HOXA4 in NCI-H1975 and NCI-H446 cells but markedly decreased in NCI-H1299 cells in which HOXA4 was silenced (Fig. 6c, d).

Further, GSK3β promoter-luciferase assays were conducted in NCI-H1975 and NCI-H446 cells in which HOXA4 had been overexpressed or silenced. The results showed that the relative luciferase activity was markedly enhanced by HOXA4 overexpression but notably reduced by HOXA4 knockdown (Fig. 6e). The results of a chromatin immunoprecipitation (ChIP)-quantitative PCR (qPCR) assay showed that the binding level of HOXA4 at the GSK3β promoter was consistent with the expression level of HOXA4 (Fig. 6f). These data confirmed that HOXA4 could bind to the GSK3β promoter and that the HOXA4 expression level affected GSK3β promoter activity and transcription.

### The Wnt signaling pathway mediates HOXA4 effects on cell growth, migration and invasion

**Fig. 7:**
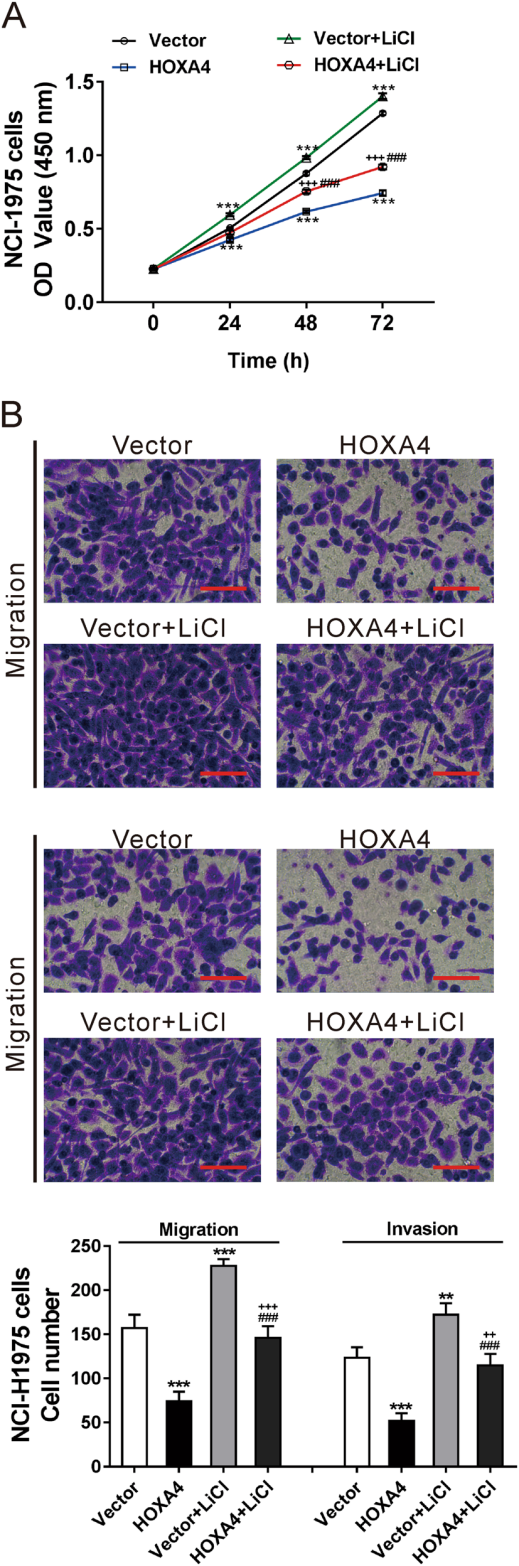
The Wnt signaling pathway mediated the effects of HOXA4 on cell growth and invasion. NCI-H1975 cells were infected with HOXA4 overexpression lentivirus or control vector lentivirus (Vector). LiCl (20 mM) was added at 24 h post viral infection. CCK-8 (**a**) and transwell assays (**b**) were performed to determine the proliferation, invasion and migration abilities of NCI-H1975 cells with LiCl treatment and HOXA4 overexpression. ***P < 0.001 vs. Vector; ^###^*P* < 0.001 vs. HOXA4; ^++^*P* < 0.01 and ^+++^*P* < 0.001 vs. Vector+LiCl

To clarify whether the Wnt signaling pathway mediates the effects of HOXA4 on cell growth, migration and invasion, NCI-H1975 cells with HOXA4 overexpression were exposed to LiCl, which activates Wnt signaling^21^ (Fig. 7). HOXA4 overexpression markedly suppressed cell growth, migration and invasion, and this effect was abolished by LiCl stimulation. These data confirmed that the Wnt signaling pathway is involved in the functions of HOXA4 on cell growth, migration and invasion.

### HOXA4 inhibits lung cancer cell tumorigenesis in vivo

**Fig. 8:**
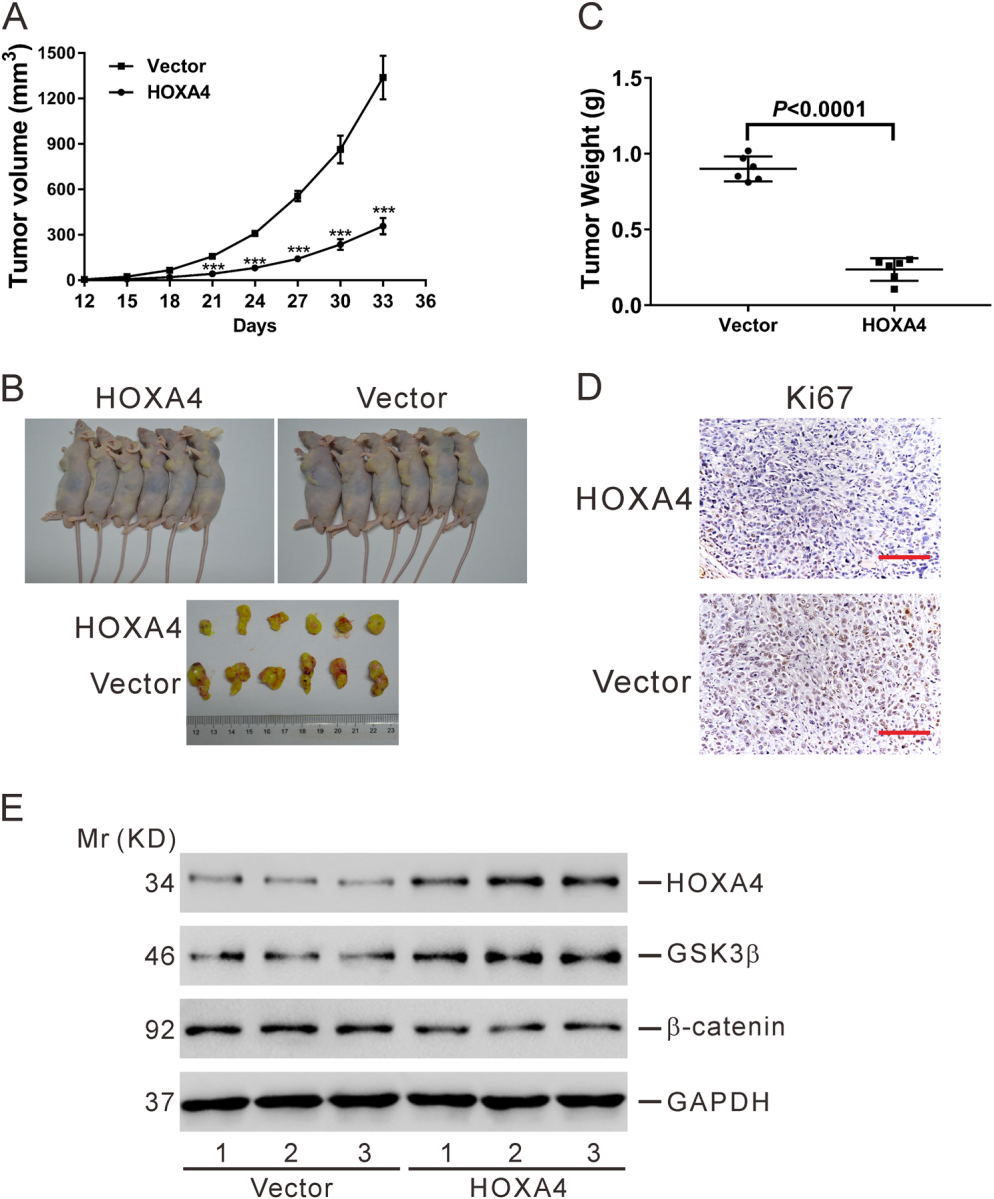
HOXA4 inhibited tumorigenesis of lung cancer cells in vivo. NCI-H1975 cells transduced with empty vector or HOXA4-overexpressing virus were injected into nude mice (*n* = 6). **a** Tumor volumes were measured every 3 days after injection. ***P < 0.001. **b**, **c** At 33 days after injection, xenograft tumors were recovered and weighed. **d** The tumor sections were stained for IHC using antibodies against Ki67. Magnification: ×200. Scale bars=100 μm. **e** Western blotting was performed to assess the protein levels of HOXA4, GSK3β, and β-catenin in xenograft tumors. Data representing three replications are shown

To determine whether HOXA4 expression could affect tumorigenesis, NCI-H1975 cells transduced with a HOXA4 overexpression virus or vector virus were transplanted into nude mice. We found that the tumor growth in the HOXA4 overexpression group was significantly slower than that in the vector group (Fig. 8a). At 33 days after inoculation, tumor size (Fig. 8b), and weight (Fig. 8c) was noticeably decreased in the HOXA4 overexpression group compared with the vector group. Moreover, Ki67-positive signals from the xenograft tumors also declined in the HOXA4 overexpression group (Fig. 8d). Western blotting analysis revealed that tumor tissues formed from HOXA4-overexpressing cells displayed higher levels of HOXA4 and GSK3β and lower levels of β-catenin than did cells in the vector group. These results suggest that HOXA4 overexpression significantly reduces the proliferation capacity of lung cancer cells and suppresses the Wnt signaling pathway in vivo.

## Discussion

HOXA4 is reportedly overexpressed in colorectal cancer^15^ and epithelial ovarian cancer^16^. The *Hoxa4* gene is involved in the patterning of the mouse lung during embryonic development^14^. We hypothesized that HOXA4 may be associated with lung carcinogenesis. To test this hypothesis, we analyzed the expression of HOXA4 in the TCGA lung cancer dataset and our own patient cohort. We found that HOXA4 levels were significantly lower in lung cancer tissues compared with normal lung tissues (Fig. 1). We also observed that HOXA4 expression in lung cancer was significantly associated with tumor size, TNM stage, lymph node metastasis and overall survival (Fig. 2 and Table 1). These findings indicated that HOXA4 can be used as a potential diagnostic and prognostic marker for lung cancer.

The functions of HOXA4 in cancer progression have been rarely studied except for its role in suppressing migration in ovarian cancer cell lines^17^. In the present study, we explored the effects of HOXA4 expression levels on the growth, migration and invasion of lung cancer cells by manipulating HOXA4 expression with lentiviral transduction (Figs. 4, 5, and 8). To our knowledge, this is the first report that HOXA4 may potentially serve as a tumor suppressor in lung cancer. We also showed that overexpression of HOXA4 significantly promoted cell apoptosis (Fig. 4c, d), suggesting that increased cell apoptosis is one of the potential reasons for the decreased proliferation observed in HOXA4-overexpressing cells.

The Wnt signaling pathway plays an important role in lung cancer tumorigenesis and prognosis^22^. Prior studies have suggested that HOXA5 represses the Wnt signaling activity in colon cancer cell lines^23^, whereas HOXA9 and HOXA10 activate Wnt signaling activity in human CD34+ umbilical cord blood cells^24^. However, it is not known whether HOXA4 can affect the Wnt signaling pathway. Here, GSEA on the TCGA dataset showed that HOXA4 expression was negatively correlated with the Wnt signaling pathway (Fig. 2c). Ectopic expression of HOXA4 led to an obvious decrease in the protein levels of downstream effectors of the Wnt pathway (β-catenin, Cyclin D1, c-Myc, and Survivin) (Fig. 6a), which are involved in the development of lung cancer. Reduced β-catenin expression is a poor prognostic factor for patients with lung cancer^25^. Genetic alteration of Cyclin D1 is a prognostic factor in NSCLC^26,27^. Suppression of c-Myc, a well-known oncogene, decreases proliferation in lung cancer cell lines^28^. Inhibition of Survivin expression increases apoptosis in NSCLC cells^29^. Our findings suggest that Wnt signaling may be involved in HOXA4 suppression-mediated tumorigenesis, although we could not exclude other factors. Further studies are needed to clarify this issue using specific knockdown of the proposed targets (β-catenin, Cyclin D1, c-Myc, and Survivin). GSK3β, a major component in the Wnt pathway, promotes the phosphorylation and degradation of β-catenin, thus inhibiting Wnt signaling^22^. Dysregulation of GSK3β has been observed in various human cancers. GSK3β may functions as a tumor suppressor^30–36^ and tumor promoter^37–41^, depending on the cell types and signaling pathways involved. For example, activation of GSK3β induces apoptosis, accumulation of β-catenin and suppression of Cyclin D1 expression in human breast cancer cells^33^. GSK3β has been proposed to be involved in breast cancer cell metastasis via regulation of Snail1 activity^36^. The overexpression of inactivated GSK3β may be a worse prognostic factor for patients with lung cancer^34^. In contrast, GSK3β has been reported to be upregulated in NSCLC tissues and to positively regulate tumor cell proliferation^38^. Here, we found that HOXA4 overexpression increased GSK3β protein and mRNA levels. The relative luciferase activity of GSK3β and enrichment of the GSK3β promoter was markedly enhanced by HOXA4 overexpression but notably reduced by HOXA4 knockdown (Fig. 6). Accordingly, we speculated that HOXA4 might increase GSK3β expression via promoting its transcription and then inhibiting Wnt signaling. Moreover, LiCl treatment, which can inactivate GSK3β, markedly promoted the cell growth and invasion of HOXA4-overexpressing cells (Fig. 7). Our data indicated that inactivation of GSK3β may promote tumorigenesis, which was consistent with a previous study^34^. Our data also indicated that the Wnt signaling pathway mediates the functions of HOXA4 regarding cell growth, migration and invasion.

In summary, down-regulation of HOXA4 was associated with poor prognosis of lung cancer. The ectopic expression of HOXA4 in lung cancer cells decreased cell proliferation, migration and invasion as well as Wnt signaling. HOXA4 is a potential tumor suppressor in lung cancer.

## Materials and methods

### Patients and tissue samples

This study was approved by the Research Ethics Committee of Shanghai Jiao Tong University Affiliated Sixth People’s Hospital. A total of 100 patients with lung cancer who underwent surgical resection at the Department of Thoracic-cardiovascular Surgery, Shanghai Jiao Tong University Affiliated Sixth People’s Hospital, were enrolled in this study. Written informed consent was obtained from all enrolled patients. Clinical information was obtained by review of medical records. The follow-up period lasted 5 years. Primary lung cancer tissues (*n* = 100) and adjacent non-cancerous tissues (*n* = 40) were obtained during surgery, immediately frozen in liquid nitrogen and stored at −80 °C until use.

### Quantitative reverse transcription-PCR (qRT-PCR)

Total RNA was isolated from cells or tissues using TRIzol reagent (Life Science, Carlsbad, CA, USA) per the manufacturer’s instructions. Total RNA was treated with DNase and then reverse transcribed to cDNA using the RevertAid First-Strand cDNA Synthesis Kit (Thermo Scientific, Rockford, IL, USA). qRT-PCR analysis was performed with SYBR Green qPCR Master Mixes (Thermo Scientific) on an ABI 7300 system (Applied Biosystem, Foster City, CA, USA). The messenger RNA (mRNA) levels of HOXA4, glycogen synthase kinase-3β (GSK3β) and GAPDH (internal control) were quantified using the following primers: HOXA4, forward: 5′- ATAACGGAGGGGAGCCTAAG-3′, reverse: 5′- GCTCAGACAAACAGAGCGTG-3′; GSK3β, forward: 5′- AAGGTTAGCTGGTAACTGTAGG -3′, reverse: 5′- TTTCTGGGTACTGGTTCACTTC -3′; GAPDH, forward: 5′-CACCCACTCCTCCACCTTTG-3′, reverse: 5′- CCACCACCCTGTTGCTGTAG-3′. All samples were run in triplicate. The mRNA levels of target genes were normalized to GAPDH mRNA using the 2^−ΔΔCt^ method^42^.

### Bioinformatics analysis

The lung cancer dataset, which included 488 lung cancer tissues and 58 normal lung tissues, was obtained from The Cancer Genome Atlas project (TCGA, https://tcga-data.nci.nih.gov/tcga/). Student’s *t*-test was then used to compare HOXA4 expression levels between lung cancer and normal lung tissues.

Gene set enrichment analysis (GSEA) was performed to identify pathways associated with HOXA4 mRNA expression levels in the TCGA lung cancer dataset, as previously described^43^. GSEA software was obtained from the Broad Institute (http://www.broad.mit.edu/gsea).

### Cell lines

NCI-H446, NCI-H1975, SPC-A-1, A549, NCI-H1299, BEAS-2B, and 293T cells were obtained from the Cell Bank of the Shanghai Biology Institute (Shanghai, China) and maintained in a 5% CO_2_ incubator at 37 °C. A549 and 293T cells were grown in DMEM, and other cells were cultured in RPMI-1640. All media were supplemented with 10% fetal bovine serum (FBS) and 1% penicillin/streptomycin (Life Technologies).

### Lentiviral production and infection

DNA oligonucleotides encoding three small hairpin RNAs (shRNAs) against HOXA4 (target sequences: shHOXA4-1, 5′- CCAACTACATCGAGCCCAA-3′, shHOXA4-2, 5′-TCCATGTCAGCGCCGTTAA-3′, shHOXA4-3, 5′-AGATGCGATCCTCCAATTC-3′), or a negative control (shNC) were inserted into the lentiviral vector pLKO.1-puro (Addgene, Cambridge, MA, USA). Human HOXA4 cDNA (synthesized by Genewiz, Suzhou, China) was inserted into the EcoRI and BamHI sites of the lentiviral vector pLVX-puro (Clontech, Palo Alto, CA, USA). For lentiviral production, 293T cells were transfected with the lentiviral vector along with packaging plasmids using Lipofectamine 2000 (Life Science) according to the manufacturer’s instructions. At 48 h and 72 h after transfection, culture media was collected, pooled and filtered. Lung cancer cell lines were infected with the indicated lentivirus, and HOXA4 expression was determined by qRT-PCR and western blotting at 48 h after infection.

### Western blotting

An antibody against HOXA4 from Abcam and anti-GSK3β, anti-β-catenin, anti-Cyclin D1, anti-c-Myc, anti-Survivin and anti-GAPDH antibodies from Cell Signaling Technology were used in western blotting analysis. Cells or tissues were lysed in RIPA buffer (Solarbio, Beijing, China) with a protease inhibitor cocktail (Sigma Aldrich, St. Louis, MO, USA). Protein concentration was evaluated with a BCA (bicinchoninic acid) protein assay kit (Thermo Scientific). Approximately 30 μg protein lysate of each sample was separated by 10% or 15% SDS–PAGE and transferred onto nitrocellulose membranes (Millipore, Bedford, MA, USA). The membranes were incubated with primary antibodies at 4 °C overnight and then with a horseradish peroxidase (HRP)-conjugated secondary antibody at room temperature for 1 h. The target proteins were detected with the enhanced chemiluminescence substrate (Millipore).

### Cell growth assay

To compare cell growth rates, 3 × 10^3^ cells in 100 μl media were seeded in 96-well plates and cultured overnight. The cells were transduced with the desired virus, and cell growth was assessed at 0, 24, 48, and 72 h after viral transduction using the Cell Count Kit-8 (CCK-8) assay (SAB biotech., College Park, MD, USA) according to the manufacturer’s instructions. Briefly, the culture media was replaced with 10% CCK-8 solution (in culture medium) and incubated at 37 °C for 1 h. Optical density (OD) at 450 nm was detected with a microplate reader (BioTek, Winooski, VT, USA). Experiments were performed in triplicate and repeated three times.

### Cell apoptosis assay

To compare cell apoptosis rates, 5 × 10^5^ cells were seeded in 6-well plates and cultured overnight. The cells were transduced with the desired virus, and cell apoptosis was assessed at 48 h after viral transduction using the Annexin V Apoptosis Detection Kit (eBioscience, San Diego, CA, USA) according to the manufacturer’s instructions. Briefly, the cells were harvested and washed in PBS. The cells were then incubated with Annexin V for 15 min followed by propidium iodide (PI) for 5 min in the dark. Cell apoptosis was analyzed in a flow cytometer (BD Biosciences, Franklin Lakes, NJ, USA). Experiments were performed in triplicate and repeated three times.

### Transwell assay

Cellular migration and invasion were determined by Transwell assay. For the migration assay, cells grown in 6-well plates were transduced with the desired virus. After 24 h, the cells were serum starved overnight. The cells were then trypsinized, 1 × 10^4^ cells in 300 μl serum-free medium were added to the upper chamber, and 700 μl media containing 10% FBS was added to the lower chamber. Following growth at 37 °C for 24 h, the migrating cells were fixed in 4% paraformaldehyde and stained with 0.5% crystal violet. The stained cells were then counted under a microscope. Experiments were performed in triplicate.

The invasion assay was performed in the same manner as the migration assay except that the upper chamber was coated with 1 mg/ml Matrigel (BD Biosciences) prior to the experiments.

### Luciferase assay

The human GSK3β promoter^44^ was amplified from genomic DNA and cloned into the KpnI and BglII sites of pGL3-basic vector (Promega, Madison, WI, USA). NCI-H1975 and NCI-H446 cells were transfected with pRL-TK (Promega) and the pGL3-GSK3β promoter using lipofectamine 2000 reagent, infected with HOXA4-expressing or vector lentivirus, or infected with HOXA4 shRNA (shHOXA4) or control shRNA (shNC). At 48 h following transfection, cells were lysed, and luciferase activity was determined using a dual-luciferase reporter assay according to the manufacturer’s protocol (Promega). Values were normalized to that of *Renilla* luciferase and then to a control.

### Chromatin immunoprecipitation (ChIP) assay

Lung cancer cells were infected with the indicated lentivirus, and ChIP assays were performed according to the manufacturer’s instructions (Abcam, Cambridge, MA, USA). Briefly, cells were washed in PBS and cross-linked with 1% formaldehyde at room temperature for 10 min. The cells were lysed and sonicated to shear DNA. Supernatant was obtained by centrifugation for 10 min at 8000 × g. Samples were added to protein A/G beads and incubated overnight with anti-GSK3β or control IgG. After washing, DNA was eluted from the immune-complex and quantitatively measured by SYBR green real-time quantitative PCR. The qPCR primers for the GSK3β promoter were 5′- GGCTTCCCAGCGTCACTTTC -3′ and 5′- ACTCGTCCTCCACCTCCTTC -3′.

### In vivo tumor xenograft studies

The animal study was performed in accordance with the instructions of the Experimental Animal Care Commission of the Shanghai Jiao Tong University Affiliated Sixth People’s Hospital. Four-to-five-week-old BALB/c nude mice (SLAC Animal, Shanghai, China; *n* = 12) were housed in specific pathogen-free (SPF) conditions on a 12-h light/dark cycle with free access to food and water. NCI-H1975 cells infected with HOXA4-expressing or vector virus were injected subcutaneously into nude mice (4 × 10^6^ cells in 100 μl PBS). Tumor size was measured every 3 days for 33 days. At 33 days after cell transplantation, the mice were killed. The xenograft tumors were resected and weighed. The tumors were subjected to western blotting and immunohistochemistry (IHC) staining.

### IHC analysis

The tumors were fixed in 10% formalin, embedded in paraffin, and cut into 5-μm-thick sections. After routine deparaffinization, rehydration and inactivation of endogenous peroxidases, the sections were immersed in 0.01 M citrate buffer (pH 6.0) and treated with microwaves for 15 min. Following incubation with 10% normal goat serum for 30 min, the sections were incubated with a Ki67 antibody (Abcam) overnight at 4 °C and then with an HRP-conjugated secondary antibody for 1 h at room temperature. The sections were stained with the 3,3-diaminobenzidine (DAB) solution (Vector Laboratories, Burlingame, CA, USA) and counterstained with hematoxylin.

### Statistical analysis

All data were analyzed using GraphPad Prism (San Diego, CA, USA). Student’s *t*-test was used to compare differences between two groups, while one-way ANOVA was used to compare differences among more than two groups. The correlation between HOXA4 expression and clinical pathological features was assessed using Fisher’s exact test. Kaplan–Meier analysis and the log-rank test were performed to compare the overall survival of patients with low or high tumor HOXA4 expression. *P* < 0.05 was considered statistically significant.
